# Association between Dietary Patterns and Atopic Dermatitis in Relation to *GSTM1* and *GSTT1* Polymorphisms in Young Children

**DOI:** 10.3390/nu7115473

**Published:** 2015-11-13

**Authors:** Jayong Chung, Sung-Ok Kwon, Hyogin Ahn, Hyojung Hwang, Soo-Jong Hong, Se-Young Oh

**Affiliations:** 1 Department of Food & Nutrition, Research Center for Human Ecology, College of Human Ecology, Kyung Hee University, 26, Kyungheedae-ro, Hoegi-dong, Dongdaemun-gu, Seoul 02447, Korea; jchung@khu.ac.kr (J.C.); kamelon@hanmail.net (S.-O.K.); ddottori@naver.com (H.A.); fullmoon0118@naver.com (H.H.); 2 Department of Pediatrics, Childhood Asthma Atopy Center, Research Center for Standardization of Allergic Diseases, University of Ulsan College of Medicine 13, Gangdong-daero, Pungnap-dong, Songpa-gu, Seoul 05535, Korea; sjhong@amc.seoul.kr

**Keywords:** dietary patterns, *GST* gene, polymorphisms, atopic dermatitis, young children

## Abstract

Previous research suggests the association of glutathione S-transferase (*GST*) gene polymorphisms or diet, but no interactions between these factors in atopic dermatitis (AD). We conducted a community-based case-control study including 194 AD and 244 matched non-AD preschoolers. Glutathione S-transferase *M1 (GSTM1*) and *T1 (GSTT1) present/null* genotypes were evaluated uisng a multiplex PCR method. We measured dietary intakes by a validated food frequency questionnaire and constructed three dietary patterns such as “traditional healthy”, “animal foods”, and “sweets” diets. In stratified analyses by *GST* genotypes, the “traditional healthy” diet and reduced AD showed association only in the *GSTM1-present* group (odd ratio (OR) 0.31, 95% confidence interval (CI) 0.13–0.75). A similar pattern of the association existed in the combined *GSTM1/T1* genotype that indicated the inverse association between the “traditional healthy” diet and AD in the double *GSTM1/T1-present* genotype group (OR 0.24, 95% CI 0.06–0.93). Results from the multiplicative test analyses showed that the “traditional healthy” diet on reduced AD was significant or borderline significant in the *GSTM1-present* group (OR 0.71, 95% CI 0.54–0.92 *vs. GSTM1-null* group) or the *GSTM1/T1* double *present* group (OR 0.63, 95% CI 0.39–1.03 *vs. GSTM1/T1* double *null* group). These findings demonstrate that the *present* type of *GSTM1* may increase susceptibility to the potential effect of the “traditional healthy” diet on AD.

## 1. Introduction

Atopic dermatitis (AD) is a chronic and relapsing inflammatory skin disease. It is one of the most common allergic diseases in children, affecting up to 25% worldwide [1]. Comparable or even greater prevalence of AD (25%–34%) has been reported in a large scale study of Korean children [2]. The majority of AD starts in early childhood, and 70% of children with AD show clinical symptoms before the age of five years [3,4]. As AD is a major health concern that severely compromises quality of life in children, understanding the factors associated with the development of AD and its prevention are critical.

Multiple genetic and environmental factors are thought to contribute to the risk and development of AD. AD is usually associated with a family history of atopic disorders, such as asthma, rhinitis, and AD itself, and twin studies have shown that the genetic contribution is substantial [5]. However, a steady increase in the prevalence of AD over recent decades indicates that environmental factors also play important roles in AD pathogenesis. Although the molecular mechanisms underlying AD are not fully understood, impaired homeostasis of oxygen/nitrogen radicals as well as increased oxidative stress have been suggested to be involved in the pathophysiology of childhood AD [6,7]. In skin inflammation associated with AD, reactive oxygen species are released during the activation and infiltration of lymphocytes, monocytes, and eosinophils [8,9].

Diet has been suggested as a predictor of health such as allergic diseases and mental health in childhood [10,11,12,13,14,15,16], although the actual association is not clear. Previously, we have demonstrated that a higher intake of dietary antioxidant vitamins, including β-carotene and vitamin E, is associated with a reduced risk of AD among preschool-age children in Korea and suggested a possible role of oxidative stress in this association [10]. Dietary pattern providing an overall view of intake draws attention because it could minimize chance inter-correlations among many nutrients in the diet. “Processed” or “Western” diets , high in fat and sugar content, or “healthy” or “prudent” diets, containing micronutrient-rich foods, have been reported regarding child mental health, although the associations are not clear [13,14,15,16].

Genetic variations in glutathione S-transferase (*GST*) that alter enzymatic activity can have a significant impact on susceptibility to diseases whose pathogenesis involves oxidative stress, as is the case in many inflammatory diseases such as atopic dematitis (AD) [17,18,19,20]. Several genetic polymorphisms have been identified in *GST* isoforms. A limited number of studies [21,22], including one of our own [23], have examined the association of *GST* gene polymorphisms with AD, yet the findings are inconsistent.

Considering that both genetic and environmental factors are important contributors to AD development, we hypothesize that interactions between genetic determinants of antioxidant capacity and diet may play a role in AD. Therefore, in the present study, we examined the association between dietary patterns and AD in relation to glutathione S-transferase M1 (*GSTM1*) and T1 (*GSTT1*)-*present/null* polymorphisms.

## 2. Methods

### 2.1. Participants and Study Design

**Figure 1:**
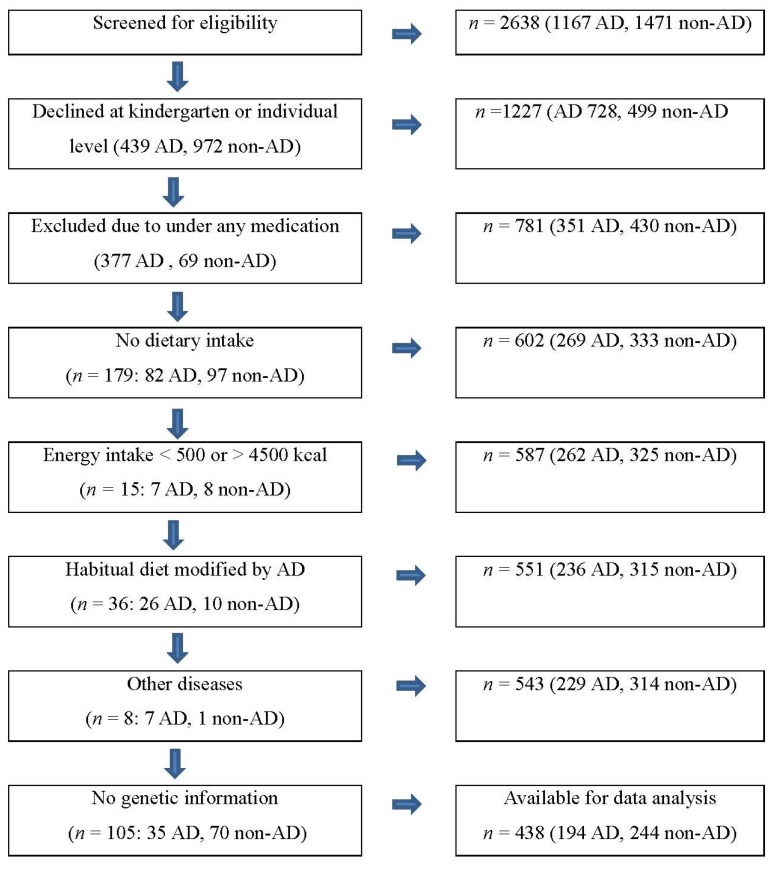
Sample selection process. Atopic Dermatitis (AD).

As shown in Figure 1, at the beginning, our participants were from a population based and matched case-control study including 781 subjects who were selected by screening eligibility from 2638 preschoolers residing in middle-income areas in large cities in Korea such as Seoul and Incheon between May and July 2006 [10,23]. We assessed the child’s AD by the Korean version of ISAAC (The International Study of Asthma and Allergies in Childhood) [10,23]. Case subjects were children who had experienced AD symptoms in the form of AD diagnosis or treatment (*n* = 351), and controls were matched by the same preschools (*n* = 430), considering both age and gender. Of those 781 participants, we excluded 343 children who had no dietary intake variables (*n* = 179; 82 AD, 97 non-AD), energy intake less than 500 kcal or greater than 4,500 kcal (*n* = 15; 7 AD, 8 non-AD), modified diet by AD (*n* = 36; 26 AD, 10 non-AD) or other diseases (*n* = 8; 7 AD, 1 non-AD), or no genetic information (*n* = 105; 35 AD, 70 non-AD). A total of 438 (194 AD, 244 non-AD) children were included in our data analyses (Figure 1). Due to the exclusion of a large number of children, we did comparison analysis including the child’s age (5.3 *vs.* 5.2 years), BMI (15.4 *vs.* 15.5) and gender (48.3% *vs.* 50.3% for girls), as well as household monthly income (38.4% *vs.* 33.8% for greater than 4 million Korean Won, close to 4000 US $). There was no significant group difference in these variables (Supplementary Table S1).

Data on dietary intake, AD, and other related information were collected by questionnaires. Blood samples were taken for the analyses of genetic information and total IgE concentration between September 2006 and January 2007.

### 2.2. Dietary Assessment

We assessed dietary intake through a validated semi-quantitative food frequency questionnaire (FFQ) used in other studies [10,24]. Reproducibility (*r =* 0.5–0.8) and validity (*r* = 0.3–0.6) of this instrument were both acceptable [10,24]. The FFQ contains 86 food items with nine non-overlapping frequency response categories as well as three portion size options (low 0.5, medium 1, high 1.5). Using CAN PRO II (Computer-Aided Nutritional Analysis Program II), developed by the Korean Nutrition Society, the amount of each food item in the FFQ was converted into grams, after which daily intakes of nutrients were calculated.

To develop dietary patterns for this study group, we used 84 food items, excluding two rarely eaten foods (organ meat and fermented salty fish). From the 84 food items, our analysis consisted of 33 food/food groups based on nutrient profiles of each food item (Table 1).

**Table 1:** Thirty-three food groups used in statistical analyses with factor analysis.

Food/Food Group	Food
Beans	Soybean curd (tofu)/curd residue, soybean (boiled with soy sauce), soymilk
Beef	Sliced beef with sauces (Galbi, Bulgogi), beef (loin, tender loin), beef soup/beef broiled down in soy
Bread	White and dark breads
Cereals	Breakfast cereals
Cheese	Cheese
Chicken	Chicken (fried), chicken (boiled, braised)
Chocolate	Chocolate
Eggs	Eggs
Fast food	Hamburger, pizza, French fries
Fats	Butter/margarine, mayonnaise
Fresh fish	White fish (pan fried, fried), white fish (grilled, broiled down in soy) blue fish (pan fried, fried), blue fish (grilled, broiled down in soy), squid/octopus, shrimps, clams/oysters
Fruit juice	Orange juice, tomato juice, other fruit juices
Fruits	Strawberries, apple, pear, mandarin/orange, tomato, banana, melon/muskmelon, watermelon, peaches/plum, grapes
Ice cream	Ice cream
*Kimchi*	Korean cabbage kimchi/seasoned cubed radish roots/young radish kimchi, other kinds of kimchi
Milk	Whole milk, flavored milk, low fat milk
*Mulchi*	Anchovy (stir-fried)
Noodles & Dumplings	Korean style noodles, spaghetti/bean sauce noodles, dumplings
Nuts	Nuts
Pork	Pork (loin, tender loin, shoulder), pork (belly)
Potatoes	Potatoes, sweet potatoes (not fried)
Processed fish	Canned tuna, fish paste
Processed meat	Ham/sausage
*Ramyeon*	*Ramyeon*
Rice	White rice, other grains
Rice cake	Rice cakes
Seaweeds	Dried laver, sea mustard
Snacks	Chips, crackers
Sweet bread	Sweet bread
Sweet drinks	Cocoa, soft drinks, sport drinks, traditional sweet drinks
Sweets	Candies, jam
Vegetables	Lettuce/cabbage (raw), lettuce/cabbage (cooked), radish, bean sprout/mungbean sprout, cucumber, spinach, perilla leaves, unripe hot pepper, onion, carrots, squash, mushrooms, roots of balloon flower/fernbrake
Yogurt	Yogurt, yogurt drinks

### 2.3. Genotyping

Genotypes for *GSTM1* and *GSTT1 present/null* polymorphisms were assessed as described by Chung *et al.* [23]. Genomic DNA was extracted from buffy coats using an AxyPrep™ Blood Genomic DNA miniprep kit (Axygen Biosciences, Union City, CA, USA), after which multiplex PCR analyses were performed. Briefly, *GSTM1*, *GSTT1*, and β-*globin* genes were simultaneously amplified by PCR along with mixed primers for each gene. PCR conditions were as follows: initial denaturation at 94 °C for 3 min, followed by 27 cycles of 94 °C for 30 s, 62 °C for 30 s, and 72 °C for 45 s, and a final extension step of 10 min at 72 °C. After amplification, PCR products were analyzed on a 2% agarose gel and stained with ethidium bromide. The presence or absence of *GSTT1* (480 bp) and *GSTM1* (215 bp) genes was determined in the presence of the control β-*globin* gene (268 bp).

### 2.4. Other Factors

Using a questionnaire, we measured household monthly income, parental education level, and child’s age as continuous variables, and parental allergic history including AD, asthma, or rhinitis, and child’s gender, nutrient supplement intake, and current exposure to smoking at home as categorical variables. Height and weight of children were measured by following recommended standard procedures [25]. Body mass index (BMI) was calculated by using height and weight measures. Serum total IgE concentrations were determined by EIA (AutoCAP system, Pharmacia, Uppsala, Sweden).

### 2.5. Statistical Analysis

Based on the 33 food/food groups (Table 1) with daily intake frequency values per 1000 kcal, we performed factor analysis to develop dietary patterns with varimax rotation [26]. Three dietary patterns were selected in accordance with the eigenvalue (>1.5), scree plots, and interpretability of factors. We calculated factor loadings for each food/food group across the three dietary factors, and a factor score for each subject obtained for the 33 food/food groups, in which intakes of food groups were weighted by their factor loadings and summed.

We named dietary patterns based on food/food groups with the most positive factor loadings. The “traditional healthy” pattern was identified by considering relatively higher intakes of vegetables, fruits, seaweeds, beans, anchovies, potatoes, fresh fish, *kimchi*, and cheese, as well as lower intake of *ramyeon*. The “animal foods” pattern was characterized by higher intakes of beef, pork, poultry, fish, and fast foods, in addition to noodles and rice cake. The “sweets” included higher intakes of fruit juice, sweet drinks, chocolate, snacks, and ice cream, but lower intake of rice (Table 2).

**Table 2:** Factor-loading matrix for defining dietary patterns by the factor analysis using 33 food or food group variables (*n* = 438).

Food/Food Groups	Traditional Healthy	Animal Foods	Sweets
Vegetables	0.62	0.13	−0.13
Fruit	0.58	−0.01	0.18
Seaweeds	0.48	0.06	−0.02
Beans	0.44	0.01	0.10
*Mulchi*	0.46	−0.03	−0.06
Potatoes	0.46	0.21	−0.04
*Kimchi*	0.38	−0.08	−0.22
Fresh fish	0.37	0.21	−0.10
*Ramyeon*	−0.35	0.29	−0.17
Noodles and dumplings	−0.21	0.52	0.03
Bread	0.04	0.49	0.12
Rice cake	0.15	0.48	−0.04
Chicken	−0.07	0.47	−0.02
Fast food	−0.19	0.43	0.19
Sweet bread	−0.03	0.40	0.06
Beef	0.15	0.37	0.04
Sweets	−0.01	0.36	0.35
Fats	0.11	0.33	−0.01
Pork	0.05	0.30	0.05
Processed meat	−0.21	0.28	0.09
Processed fish	0.09	0.25	0.01
Milk	0.01	−0.34	0.19
Fruit juice	0.24	0.01	0.50
Sweet drinks	−0.23	0.17	0.46
Chocolate	−0.14	0.14	0.46
Snacks	−0.28	0.14	0.41
Ice cream	0.02	0.06	0.38
Cheese	0.32	−0.13	0.33
Rice	−0.23	−0.01	−0.60
Yogurt	0.20	−0.23	0.29
Eggs	0.16	0.15	−0.27
Nuts	0.27	−0.05	0.27
Cereals	−0.04	0.02	0.16

Each dietary pattern was divided into high (Q4) and low (Q1–Q3) groups according to the quartiles of dietary pattern scores. In addition to initial crude models, multivariate logistic regression models were used to estimate the effects of dietary patterns and *GST* genotypes on AD. As potential residual confounders, parental allergic history, maternal education level, household income, and child’s age, gender, BMI, total energy and nutrient supplement intakes, and secondary smoking exposure had been considered. Among these variables, household income, maternal education level, and child’s secondary smoking exposure were excluded in the analytic model because these variables did not show any significant difference between AD and non-AD groups.

To investigate the association between dietary patterns and AD with respect to *GST* genotypes, stratified analyses were performed after dividing the subjects into two groups for the *GSTM1* and *GSTT1* (*null* and *present*), and three groups for the *GSTM1/T1* (double *null*, either *present*, or double *present*). The stratified analysis by *GST* genotype was conducted adjusted for the confounders in the corresponding model. Multiplicative interactions were performed using the corresponding models that included the interaction term to examine the modifying effect of *GST* genotypes on the association between dietary patterns and AD. Results were reported as odds ratios (OR) and 95% confident intervals (CI). Significance was set at *p* < 0.05. Statistical analyses were conducted with SAS version 9.3 (SAS Institute Inc., Cary, NC, USA).

### 2.6. Ethics Statement

This study was conducted in accordance with the guidelines detailed in the Declaration of Helsinki, and all procedures involving human subjects were approved by the Institutional Review Board of the College of Human Ecology at Kyung Hee University [10,23]. Written informed consent was obtained from all parents of participating children.

## 3. Results

When we compared the high (Q4) and low (Q1–Q3) groups of dietary patterns, the “traditional healthy” diet was associated with higher intakes of protein, unsaturated fat, and micronutrients (Table 3). In particular, higher intakes of β–carotene and vitamin C (2.1 and 1.5-fold difference between the high and low groups, respectively) were substantial. The “animal foods” dietary pattern showed higher intakes of macronutrients except for plant protein and saturated fatty acids (SFA), but had no associations with micronutrients excluding vitamin E. The “sweets” diet was relevant to higher intakes of energy, plant fat, SFA, retinol, and vitamin C, as well as a lower intake of plant protein.

When we compared nutrient intakes by AD, AD showed an association with lower intakes of vitamin E, folate, and possibly β-carotene (Table 4). General characteristics were similar between AD and non-AD children except for child’s total IgE concentration and allergic history of parents (Table 4). In the AD group, the majority of children (80.4%) showed experience of physician’s diagnosis of AD, followed by AD symptoms (45.8%–49.5%) and AD treatment (22.6%).

The proportions of children with the *null* genotype in *GSTM1* and *GSTT1* were close to 60% and 50%, respectively (Table 5). When the *GSTM1* and *GSTT1* genotypes were combined, about 30% of children carried the *null* genotype for both genes. There were no associations of AD with *GST* genotypes and dietary patterns in univariate analyses.

In stratified analyses by *GST* genotypes (Table 6), the “traditional healthy” diet and reduced AD showed association only in the *GSTM1-present* group (OR 0.31, 95% CI 0.13–0.75). A similar pattern of the association existed in the combined *GSTM1/T1* genotype, which indicated an inverse association between the “traditional healthy” diet and AD in the double *GSTM1/T1-present* genotype group (OR 0.24, 95% CI 0.06–0.93). There was a stronger association between the “traditional healthy” diet and AD (7%) in the *GSTM1/T1* double *present* group (OR 0.24) than the case of *GSTM1-present* (OR 0.31) group. Results from the multiplicative test analyses showed that the “traditional healthy” diet on reduced AD was significant or borderline significant in the *GSTM1-present* group (OR 0.71, 95% CI 0.54–0.92 *vs. GSTM1-null* group) or the *GSTM1/T1* double *present* group (OR 0.63, 95% CI 0.39–1.03 *vs. GSTM1/T1* double *null* group). These associations did not exist in the “animal foods” and “sweets” dietary patterns.

**Table 3:** Associations of daily nutrient intakes with dietary patterns between the low (Q1–Q3, *n* = 329) and high (Q4, *n* = 109) groups *.

Nutrient	Traditional Healthy					Animal Foods					Sweets				
	Low (Q1–Q3)		High (Q4)		*p*	Low (Q1–Q3)		High (Q4)		*p*	Low (Q1–Q3)		High (Q4)		*p*
	Mean	SE	Mean	SE		Mean	SE	Mean	SE		Mean	SE	Mean	SE	
Energy (kJ)	1556.3	37.1	1511.9	64.5	0.551	1583.0	37.0	1431.4	64.2	0.041	1497.5	36.8	1689.3	64.0	0.010
Animal protein (g)	32.0	0.6	39.3	1.1	<.0.001	32.8	0.6	36.8	1.1	0.003	33.9	0.7	33.4	1.1	0.706
Plant protein (g)	22.6	0.3	23.8	0.5	0.048	23.1	0.3	22.5	0.5	0.363	23.7	0.3	20.5	0.5	<0.001
Animal fat (g)	28.0	0.6	32.6	1.1	<0.001	28.4	0.6	31.5	1.1	0.011	28.7	0.6	30.5	1.1	0.140
Plant fat (g)	18.5	0.4	20.8	0.7	0.004	18.0	0.4	22.4	0.7	<0.001	17.9	0.4	22.8	0.7	<0.001
Vitamin A (μg, RE)	413.6	10.3	652.4	17.8	<0.001	481.1	11.7	448.7	20.4	0.170	459.9	11.7	512.8	20.4	0.025
Retinol (μg)	229.5	5.7	258.5	9.9	0.012	240.8	5.7	224.4	10.0	0.157	216.3	5.4	298.2	9.5	<0.001
β-carotene (μg)	1247.3	55.0	2561.9	95.6	<0.001	1598.4	63.4	1502.3	110.4	0.452	1560.3	63.5	1617.3	110.7	0.656
Vitamin C (mg)	65.3	2.3	97.3	4.0	<0.001	73.8	2.4	71.6	4.2	0.659	65.6	2.3	96.4	4.0	<0.001
Folate (μg)	172.1	2.8	231.4	4.9	<0.001	188.7	3.1	181.3	5.5	0.242	186.6	3.2	187.8	5.5	0.855
Vitamin E (mg, α-TE)	8.5	0.2	12.3	0.4	<0.001	8.7	0.2	11.6	0.4	<0.001	9.5	0.2	9.2	0.4	0.513
Saturated fatty acids (g)	12.1	0.3	13.1	0.6	0.131	12.5	0.3	11.9	0.6	0.326	11.9	0.3	13.8	0.6	0.004
Monounsaturated fatty acids (g)	8.0	0.2	9.7	0.4	<0.001	8.1	0.2	9.4	0.4	0.003	8.3	0.2	8.8	0.4	0.224
Polyunsaturated fatty acids (g)	3.9	0.1	5.5	0.2	<0.001	4.0	0.1	5.2	0.2	<0.001	4.3	0.1	4.2	0.2	0.749

Abbreviations: SE = standard error, RE = retinol equivalents, TE = tochopherol equivalents; * Adjusted for energy intake except for the energy variable.

**Table 4:** Associations of nutrient intakes and general characteristics with atopic dermatitis (AD) in young children.

	AD (*n* = 194)		Non-AD (*n* = 244)		*p* *
	Mean	SD	Mean	SD	
Age (year)	5.4	1.2	5.3	1.3	0.270
BMI (kg/m^2^)	18.3	3.5	17.9	3.4	0.260
Birth weight (kg)	3.3	0.7	3.3	0.8	0.876
Daily nutrient intake					
Energy (kJ)	6305.2	2622.3	6592.8	2957.5	0.289
Animal protein (g)	31.8	18.6	35.4	23.3	0.073
Plant protein (g)	22.4	9.4	23.3	10.2	0.324
Animal fat (g)	27.5	16.8	30.5	21.0	0.095
Plant fat (g)	18.2	10.5	19.8	11.9	0.130
Vitamin A (μg RE)	442.5	274.3	497.3	321.0	0.055
Retinol (μg)	231	158.8	241.2	161.8	0.509
β-carotene (μg)	1442.2	1244.3	1679.6	1397.8	0.065
Vitamin C (mg)	69.3	57.1	76.4	61.8	0.217
Folate (μg)	173.8	88.0	197.3	106.5	0.012
Vitamin E (mg α-TE)	8.5	5.3	10.2	7.4	0.005
Saturated fatty acids (g)	11.9	7.8	12.7	9.1	0.357
Monounsaturated fatty acids (g)	7.8	4.6	8.8	6.8	0.072
Polyunsaturated fatty acids (g)	3.9	2.4	4.6	3.5	0.009
Total IgE (U/mL) ^†^	325.8	617.2	187.7	307.9	0.006
	*n*	(%)	*n*	(%)	
Gender (boys)	103	(53.4)	123	(50.4)	0.539
AD status ^‡^					
Symptoms (*n* = 192)	88	45.8			
Itchy rash in the last year (*n* = 180)	89	49.5			
Diagnosis by physician (*n* = 194)	156	80.4			
Treatment (*n* = 190)	43	22.6			
Current exposure to smoking at home	31	(17.0)	35	(15.4)	0.645
Supplementary multivitamin use	80	(46.0)	110	(48.9)	0.564
Household income (10^4^ Won/mo) ^§^					
<200	28	(15.0)	30	(12.7)	0.733
200–399	90	(48.1)	113	(47.7)	
≥400	69	(36.9)	94	(39.7)	
Maternal educational level (≥16 year)	86	(44.8)	99	(40.7)	0.396
Allergic history of mother					
Asthma	9	(4.6)	13	(5.3)	0.743
Rhinitis	46	(23.7)	57	(23.4)	0.932
AD	26	(13.4)	14	(5.7)	0.006
Allergic history of father					
Asthma	9	(4.6)	6	(2.5)	0.213
Rhinitis	47	(24.2)	35	(14.3)	0.008
AD	22	(11.3)	17	(7.0)	0.110

Abbreviations: RE = retinol equivalents, TE = tochopherol equivalents; * Unadjusted; ^†^ *n* = 182 for AD, *n* = 232 for Non-AD; ^‡^ Only for AD group; ^§^ Approximately 10^4^ Won =10 US dollars.

**Table 5:** Associations of the glutathione S-transferase *M1* (*GSTM1*) and *T1* (*GSTT1*) genotypes and dietary patterns with atopic dermatitis (AD).

	AD		Non-AD		p *
	*n*	%	*n*	%	
Genotypes					
*GSTM1*					0.466
*Null*	118	60.8	140	57.4	
*Present*	76	39.2	104	42.6	
*GSTT1*					0.686
*Null*	98	50.5	128	52.5	
*Present*	96	49.5	116	47.5	
*GSTM1/GSTT1*					0.638
Double *null*	57	29.4	75	30.7	
Either *null*	102	52.6	118	48.4	
Double *present*	35	18.0	51	20.9	
Dietary patterns					
Traditional healthy					0.466
Low (Q1-Q3)	149	45.3	180	54.7	
High (Q4)	45	41.3	64	58.7	
Animal foods					0.873
Low (Q1-Q3)	145	44.1	184	55.9	
High (Q4)	49	45.0	60	55.0	
Sweets					0.776
Low (Q1-Q3)	147	44.7	182	55.3	
High (Q4)	47	43.1	62	56.9	

* Unadjusted.

**Table 6:** Association between dietary pattern and atopic dermatitis (AD) by of the glutathione S-transferase *M1* (*GSTM1*) and /or *T1* (*GSTT1*) genotypes (*n* = 438) *.

	AD *vs.* Non AD					
	aOR	95% CI	*p* for Chi-Square ^†^	aOR	95% CI	*p* for Interaction ^‡^
Tradition healthy						
*GSTM1*						
*Null*	1.24	(0.68, 2.26)	0.495	1		
*Present*	0.31	(0.13, 0.75)	0.009	0.71	(0.54, 0.92)	0.011
*GSTT1*						
*Null*	0.67	(0.34, 1.30)	0.239	1		
*Present*	0.90	(0.45, 1.81)	0.788	1.08	(0.85, 1.37)	0.542
*GSTM1/GSTT1*						
Double *null*	0.93	(0.41, 2.13)	0.881	1		
Either *null*	0.97	(0.49, 1.90)	0.931	1.27	(0.90.1.37)	0.176
Double *present*	0.24	(0.06, 0.93)	0.039	0.63	(0.39, 1.03)	0.065
Animal foods						
*GSTM1*						
*Null*	1.07	(0.56, 2.08)	0.843	1		
*Present*	1.25	(0.62, 2.52)	0.550	1.04	(0.82, 1.32)	0.760
*GSTT1*						
*Null*	1.07	(0.55, 2.07)	0.861	1		
*Present*	1.18	(0.60, 2.32)	0.649	1.03	(0.81, 1.30)	0.836
*GSTM1/GSTT1*						
Double *null*	1.18	(0.47, 2.98)	0.741	1		
Either *null*	0.95	(0.49, 1.84)	0.891	0.88	(0.64, 1.21)	0.423
Double *present*	1.68	(0.60, 4.68)	0.329	1.17	(0.79. 1.72)	0.441
Sweets						
*GSTM1*						
*Null*	0.68	(0.36, 1.26)	0.220	1		
*Present*	1.53	(0.73, 3.20)	0.266	1.23	(0.96, 1.56)	0.098
*GSTT1*						
*Null*	0.85	(0.42, 1.73)	0.665			
*Present*	1.02	(0.54, 1.93)	0.963	1.05	(0.82,1.33)	0.712
*GSTM1/GSTT1*						
Double *null*	0.59	(0.24, 1.44)	0.247	1		
Either *null*	1.04	(0.52, 2.10)	0.920	1.02	(0.74, 1.41)	0.897
Double *present*	1.62	(0.61, 4.33)	0.343	1.27	(0.87, 1.86)	0.212

* Model included the main and interaction effects of the *GSTM1* and/or *GSTT1* genotype (*present vs. null*) and dietary pattern (high *vs.* low) with adjustment for child’s age, sex, total energy intake, multivitamin use and BMI, and parental history of allergic diseases; **^†^** *P* for the stratified association between dietary patterns and AD by *GST-null* and *present* groups in the corresponding models; **^‡^** *P* for the multplicative association between AD and dietary pattern (high *vs.* low) in the *GST present* group compared to this association in the *GST null* group as reference (OR 1) in the corresponding models. *N null*; *p present.*

## 4. Discussion

Our findings suggest the association between the “traditional healthy” diet and reduced AD in children with the *GSTM1-present* genotype, and that children with *GSTT1-present* genotype may be more susceptible to this association. Such associations may not be relevant to an inactive *GST* allele such as the *GST-null* genotype.

Despite a lack of clear explanation, the absence of an association of the *GSTM1-* or *GSTT1-null* genotype with dietary components may suggest that certain polyphenols and carotenoids induce the expression of *GST* genes and increase the activities of *GST* enzymes [27,28,29]. Thus, the lack of an active *GST* allele would inhibit the response to dietary components that affect the expression of *GST* genes. Similar to our findings, a recent human intervention study [30] reported that a high-fruit juice and vegetable diet significantly increased erythrocyte *GST* activities and antioxidant capacity-related biomarker levels in blood from *GSTM1-* and *GSTT1-present* participants, whereas blood from *GSTM1-* and *GSTT1-null* participants was unaffected. Even though the interactions of *GST* genotypes and diet in relation to AD have not been studied yet, previous reports [31,32] that examined these interactions in other diseases support our findings. Specifically, a significant inverse association between intake of carotenoid-rich or cruciferous vegetables and cancer risk was detected in carriers of the *GSTM1-* or *GSTT1-present* genotype but not in those participants with the *GSTM1-* or *GSTT1-null* genotype. These results, as well as our findings, suggest that carriers of the *GSTM1-* or *GSTT1-present* genotype may be more responsive to dietary changes and could benefit more from healthy dietary patterns.

There have been conflicting reports on the association of *GST present/null* genotypes with AD [21,22,33]. Consistent with our results, the *GSTM1-present* genotype has been reported to be relevant to AD only in the presence of specific environmental stimuli (prenatal smoke exposure) [34]. Detection of these gene-diet interactions suggests that it is important to determine the effects of gene polymorphisms as well as appropriate dietary information in order to account for the heterogeneity of previous findings. Further, unlike genetic determinants, diet can be modified by lifestyle intervention. Therefore, understanding the interactions between genetic and dietary factors is valuable in establishing dietary guidelines to reduce disease risk.

Several studies [10,35,36,37,38] have shown the association between nutrient/food intakes and AD, but little is known regarding specific dietary patterns relevant to AD in relation to *GST* genes. Dietary pattern analysis considers the effects of the whole diet, including interactions and synergistic effects among nutrients and foods [39]. As nutrients and foods are not consumed in isolation, dietary patterns more accurately reflect eating habits than nutrient intake levels. The “traditional healthy” pattern that showed a positive impact on reduced AD in this study is somewhat similar to the “Mediterranean diet”, which mainly involves higher intakes of micronutrients [40]. Adherence to a “Mediterranean diet” during pregnancy has been demonstrated as a protective factor against atopy until an age of 6.5 years (OR 0.55, 95% CI 0.31–0.97) [41]. On the other hand, no association between the “Mediterranean diet” and AD has been reported in 6 to 7-year-old school children in Spain [42]. This discrepancy may be explained in part by a function of effect modifiers, like *GST* genes that we found in this study.

The limitations of this study are as follows. We assessed AD based on subjective measures without considering objective indices, although the ISAAC questionnaire used in this study is an internationally standardized protocol and has been widely used to determine the prevalence of allergic diseases in Korea [2,10,43]. Another limitation would be small sample size to identify a diet-gene interaction against AD. We determined sample size at the beginning of this study based on antioxidant nutrients and AD, but with little consideration of genetic characteristics. Possibly meaningful, but unmeasured or unexamined variables such as heavy metals [44] and other genes may also limit our findings [45,46].

Regardless of these limitations, this study of gene-diet interaction in AD is meaningful because it is the first study to suggest the importance of genetic susceptibility that could play a crucial role in the “traditional healthy” diet on a child’s AD. A large scale prospective study is needed to confirm these findings.

## 5. Conclusions

We conclude that healhty diet may reduce AD only in children with the *GSTM1-present* genotype. These findings suggest genetic susceptibility to the association between diet on AD.

## Supplementary Materials

Supplementary materials can be accessed at: http://www.mdpi.com/2072-6643/7/11/5473/s1.
