# Supplementary material for: Association between Dietary Patterns and Atopic Dermatitis in Relation to GSTM1 and GSTT1 Polymorphisms in Young Children
Source: Nutrients. 2015 Nov 13;7(11):9440–52. doi: 10.3390/nu7115473 (PMC4663601; doi:10.3390/nu7115473)
Supplement: Supplementary file 1 [file nutrients-07-05473-s001.docx]

**Supplementary Materials: Association between Dietary Patterns and Risk of Atopic Dermatitis in Relation to GSTM1 and GSTT1 Polymorphisms in Young Children**

Jayong Chung ^1^, Sung-Ok Kwon ^1^, Hyogin Ahn ^1^, Hyojung Hwang ^1^, Soo-Jong Hong ^2^ and Se-Young Oh ^1,^*

**Table S1.** Comparison of general characteristics between included and excluded children in data analyses.

|  | **Included** | | | **Excluded** | | | |
| --- | --- | --- | --- | --- | --- | --- | --- |
|  | ***N*** | **Mean (%)** | **SD** | ***N*** | **Mean** | **SD** | ***P*** |
| Age (year) | 438 | 5.3 | 1.5 | 301 | 5.2 | 1.8 | 0.261 |
| BMI | 437 | 15.4 | 1.7 | 202 | 15.5 | 1.8 | 0.460 |
| Energy (kcal) | 438 | 1545.3 | 672.7 | 182 | 1407.5 | 1079.1 | 0.111 |
| Sex (female) | 211 | (48.3) |  | 151 | (50.3) |  | 0.585 |
| Income | 163 | (38.4) |  | 98 | (33.8) |  | 0.427 |

**Table S2.** Univariate associations between dietary patterns and atopic dermatitis (AD) by *GSTM1* and/or *GSTT1* genotypes in children *(n* = 438)

|  | **Traditional healthy** | | | **Animal foods** | | | **Sweets** | | |
| --- | --- | --- | --- | --- | --- | --- | --- | --- | --- |
|  | OR | **95% CI** | ***P*** | **OR** | **95% CI** | ***P*** | **OR** | **95% CI** | ***P*** |
| ***GSTM1*** | | | | | | | | | |
| Null | 1.21 | (0.70, 2.11) | 0.491 | 0.95 | (0.53, 1.72) | 0.875 | 0.71 | (0.40, 1.25) | 0.232 |
| Present | 0.44 | (0.20, 0.95) | 0.035 | 1.18 | (0.61, 2.27) | 0.624 | 1.42 | (0.72, 2.83) | 0.315 |
| ***GSTT1*** | | | | | | | | | |
| Null | 0.78 | (0.43, 1.44) | 0.430 | 0.93 | (0.51, 1.71) | 0.825 | 0.99 | (0.53, 1.86) | 0.971 |
| Present | 0.93 | (0.49, 1.77) | 0.835 | 1.16 | (0.62, 2.17) | 0.641 | 0.89 | (0.48, 1.63) | 0.696 |
| ***GSTM1/GSTT1*** | | | | | | | | | |
| Double null | 1.00 | (0.47, 2.16) | 0.993 | 0.91 | (0.40, 2.10) | 0.825 | 0.76 | (0.34, 1.69) | 0.500 |
| Either null | 1.00 | (0.54, 1.84) | 0.991 | 0.96 | (0.52, 1.76) | 0.895 | 0.93 | (0.49, 1.76) | 0.816 |
| Double present | 0.34 | (0.10, 1.14) | 0.081 | 1.49 | (0.57, 3.90) | 0.418 | 1.38 | (0.54, 3.50) | 0.499 |
